# Is Virtual Cognitive Stimulation Therapy the Future for People with Dementia? An Audit of UK NHS Memory Clinics During the COVID-19 Pandemic

**DOI:** 10.1007/s41347-023-00306-5

**Published:** 2023-02-24

**Authors:** Emily Fisher, Danielle Proctor, Luke Perkins, Cerne Felstead, Joshua Stott, Aimee Spector

**Affiliations:** 1 Research Department of Clinical, Educational and Health Psychology, University College London, Gower Street, London, WC1E 6BT UK; 2 Central and North West London NHS Foundation Trust, London, UK

**Keywords:** Cognitive stimulation therapy, Dementia, COVID-19, Psychosocial interventions, Digital technology, Telemental health

## Abstract

Access to psychosocial interventions for people with dementia, such as Cognitive Stimulation Therapy (CST), has been restricted during the COVID-19 pandemic. Some services have shifted to provision via videoconferencing, but the prevalence of this is unknown. This audit aimed to understand provision of virtual CST (vCST) within National Health Service (NHS) memory clinics throughout the UK and Channel Islands and investigate plans for ongoing CST provision. A cross-sectional survey was circulated to NHS memory clinics, which included closed and open-ended questions to generate quantitative and qualitative data. Thirty-three memory clinics responded to the survey. During the pandemic, 55% of respondents offered vCST, whereas 45% offered no CST. Of those offering vCST, 80% plan to continue with a hybrid model of separate face-to-face and vCST groups, whilst 20% intend to deliver face-to-face CST only. Reported positive aspects of vCST were participant and staff enjoyment, perceived improved digital confidence in participants, and improved accessibility for those who cannot attend face-to-face groups. Negative aspects related to digital poverty, limited digital literacy, support needed from carers, the impact of sensory impairment on engagement, and staff time commitment. Virtual CST has been a feasible alternative to face-to-face services during the pandemic but should not completely replace in-person groups. A hybrid approach would increase accessibility for all. Future research should explore efficacy of vCST and seek to understand patterns of exclusion from such digital interventions.

**Supplementary Information:**

The online version contains supplementary material available at 10.1007/s41347-023-00306-5.

## Introduction

During the COVID-19 pandemic, public health measures in the UK included national lockdowns and restrictions on face-to-face healthcare services. The measures in place varied from March 2020 until August 2021, but for the majority of this period, access to face-to-face psychosocial interventions for people with dementia has been restricted (Giebel et al., 2021). As a result, many services shifted to delivery through digital technology, using videoconferencing platforms such as Zoom or Skype (Cuffaro et al., 2020). Delivery of psychosocial interventions through videoconferencing for people with dementia is a relatively new, but expanding, field of interest. Previous studies in this area include delivery of goal-orientated cognitive rehabilitation (Burton & O’Connell, 2018) and a weekly psychosocial support and psychoeducational intervention for people with dementia and their carers (Lai et al., 2020). Both found that virtual delivery was both feasible and resulted in outcomes comparable to face-to-face delivery. However, there are well-documented inequalities in access to digital technology for those with dementia and older people in general, including declining cognitive ability and independent day-to-day functioning, lower computer self-efficacy, and lack of trust in digital interventions (Charness & Boot, 2009; Pywell et al., 2020). Despite these barriers, the move to digital delivery has highlighted an existing gap in service provision for people who were not able to access face-to-face services outside of the pandemic context. This includes those with reduced mobility, who cannot readily access transport, and those living in rural communities (Cuffaro et al., 2020).

The National Health Service (NHS) is the publicly funded healthcare system in the UK. NHS memory clinics usually give access to specialist multi-disciplinary teams who carry out comprehensive assessment of memory problems. If a diagnosis is given, some services will provide ongoing support to people with dementia and their carers. This most commonly includes access to Cognitive Stimulation Therapy (CST), a cost-effective intervention shown to improve cognition for people with mild to moderate dementia (Lobbia et al., 2019). CST typically consists of 14, 45-min sessions over 7 weeks, involving themed group activities, which stimulate memory and language skills. CST is the only non-pharmacological intervention recommended by the National Institute of Clinical Excellence (NICE) for people with dementia to improve cognition, independence, and wellbeing (National Institute for Health & Care Excellence, 2018). Prior to the pandemic, in-person CST was actively being delivered by 90% of NHS memory clinics (Royal College of Psychiatrists, 2016). The shift to virtual delivery of CST through videoconferencing platforms as a result of the pandemic has been reported elsewhere (Cheung & Peri, 2021); however, the impact of the pandemic on CST provision within the NHS is unknown. Therefore, the aims of this audit are to.

1. Explore the provision of virtual CST within NHS memory clinics during the COVID-19-pandemic.
2. Investigate the perceived benefits and challenges of virtual CST, as well as the longer-term plans for virtual and face-to-face CST provision within NHS memory clinics.

## Methods

### Design

A mixed-method cross-sectional survey was carried out to gather information on vCST provision within NHS memory clinics.

### Recruitment

The survey was circulated to the mailing list of the Royal College of Psychiatry Memory Service National Accreditation Programme (MSNAP), a quality improvement and accreditation network for memory services the UK. The 85 memory clinics on the MSNAP mailing list were contacted. To reach services outside of the MSNAP network, the survey was also circulated to the 564 members of the dementia sub-group of the Contacts, Help, Advice & Information Network (CHAIN). This is an online support network for individuals working in health and social care, some of whom will have links to memory services. Survey respondents were staff at the memory clinics who were involved in delivery of CST.

### Procedure

Staff from MSNAP and CHAIN sent an email newsletter to their members, which included an easily accessible link to the online survey. Details for the research team were included so that staff could contact them with any questions. Responses were collected between April 14 and May 27, 2021 using the Opinio platform.

The survey consisted of a mixture of nine closed and open-ended style questions in order to generate both quantitative and qualitative data (see Appendix 1). The survey questions were developed by a Professor of Old Age Clinical Psychology and Research Assistant, both with experience of CST delivery in memory clinics. Initial questions were drafted which were refined through discussion. Staff were asked questions on CST provision prior to the COVID-19 pandemic, vCST provision during the COVID-19 pandemic, their experience of virtual CST services as service providers, and any feedback received from people with dementia and their carers. The survey also enquired as to how memory clinics plan to continue with vCST in the future.

### Analysis

All survey responses were exported onto Microsoft Excel for data cleaning and removal of duplicated information. Descriptive statistics were generated using Excel. For the qualitative feedback, researchers followed a thematic analysis approach (Braun & Clarke, 2006). Open-ended questions and responses were uploaded from Excel onto NVivo 12 software for analysis. Thematic analysis was used to identify themes inductively, using a semantic approach. Two reviewers (E. F. and D. P.) familiarised themselves with the data, and independently decided upon initial codes, which they grouped into themes and sub-themes that were finalised following discussion between the reviewers. Any discrepancies were resolved through discussion between the two reviewers. Both reviewers had clinical experience within a memory clinic and had previously attended CST groups. This prior knowledge and experience may have influenced interpretation of feedback, but reviewers regularly revisited the data to ensure that themes were focused on the explicit content of the data.

### Ethics Statement

This study was performed in line with the principles of the Declaration of Helsinki. As a service evaluation to define or judge current care, ethical approval was not required from a research ethics committee.

### Consent Statement

Consent for the service evaluation was implied through the completion of the survey. No participant personal details or contact details were obtained, and participants were asked not to provide any identifying information other than the name of the memory service.

## Results

Overall, 41 responses were received. Five responses were from duplicate memory clinics; any additional feedback was merged with the original response and the duplicate responses were then removed. Three responses without the name of the memory clinic included were also removed. A total of 33 responses, from 26 NHS trusts across 20 counties in England, Wales, Scotland, Northern Ireland, and the Channel Islands, were analysed by reviewers.

Pre-pandemic, 31 of the 33 respondents (94%) were offering face-to-face CST to their service users. When restrictions on face-to-face contact with vulnerable persons were put into place, 18 out of 33 (55%) memory clinics began offering CST virtually. Fifteen respondents (45%) were offering neither face-to-face nor vCST during the pandemic (see Table 1).

**Table 1:** CST provision in NHS memory clinics before and during the COVID-19 pandemic

**CST provision before and during the COVID-19 pandemic**			
	Yes; *n* (%)	No; *n* (%)	Total
Face-to-face CST before COVID-19	31 (94%)	2 (6%)	33
Virtual CST since COVID-19	18 (55%)	15 (45%)	33

Digital platforms used to provide vCST differed between services. Eleven memory clinics were using Microsoft Teams, whereas two were using Zoom, and one was using WebEx. Four memory clinics were offering individual CST over the telephone. Group sizes ranged between 3 and 15 members per session, although six memory clinics were offering individual CST to service users, either via the telephone or videoconferencing calls.

### Plans for CST in the Future

Eighteen respondents reported that they have been offering CST virtually during the pandemic. However, three respondents did not answer questions regarding their future service plans for CST. Of the 15 respondents with complete data, 12 out of 15 (80%) planned to continue with a hybrid model of separate face-to-face and vCST groups in future, whilst 3 out of 15 (20%) planned to deliver face-to-face CST only. None of the services reported that they would continue with vCST only.

### Qualitative Feedback

Two main themes were identified by the reviewers during analysis of the responses: (1) benefits of offering virtual CST services and (2) challenges of offering virtual CST services. These themes, their sub-themes, and example quotations are included in Tables 2 and 3.

**Table 2:** Theme: benefits of offering virtual CST services

**Sub-themes**	**Example quotations**
Accessibility of groups	‘Once set up though routines have moved well to online and the issues with physical groups of transport, traffic, environmental have all abated as people are in their own home’. (Clinic [C] 30).
	‘Virtual group sessions are an advantage because some clients are unable to transport to a venue, this ensures that the group is made available to a wider audience’. (C 13).
	‘Better for some people where transport is an issue’. (C 15).
	‘We see many benefits, including immediate access and reduced transport difficulties’. (C 19).
Engagement between person with dementia and others	‘Some of our clients have difficulties accessing face to face groups. It would be good to have a virtual option in the long term which could be supported from any of our services and would not need to be geographically co-located’. (C 23).
	‘[People] value the opportunity to “'meet'” others and it provides a focus and structure to their week when they are doing little else’. (C15).
	‘Good feedback from five groups members as they enjoyed the themes and social aspect.’. (C 16).
Positives outcomes for person with dementia	‘The feedback has been predominantly positive from participants and carers, who make reference to benefits such as: reduced social isolation, increased social stimulation/connection and enhanced cognitive “alertness”’. (C 19).
	‘Some lovely feedback [from people with dementia and carers] on people gaining confidence, improving language skills, and taking ownership of their sessions, asking to do them on their own as feeling they don’'t need that additional support’. (C 11).
Improved digital confidence for person with dementia	‘As time has gone on — people appear more confident using virtual mediums’. (C 15).
	‘Difficult to get people to try but saw benefits for people who then used technology to interact with loved ones’. (C 20).
Staff enjoyment	‘The value this has added back to the healthcare support worker role when they have been unable to work as therapeutically during the pandemic should not be underestimated’. (C 11).
	‘Really helpful to [learn] vCST skill’. (C 16).
	‘Staff here were initially nervous using remote methods, but now there is a real buzz about the sessions which is so positive after a long and tough 2020’. (C 11).

**Table 3:** Theme: challenges of offering virtual CST services

**Sub-themes**	**Example quotations**
Lack of knowledge or access to technology	‘Main issues are easily accessible hardware and internet on the client side (high levels of deprivation in the area)’. (Clinic [C] 23).
	‘Media poverty is an issue in our area and there are a lot of patients who will need face to face in future due to a lack of technology or sensory problems that make online engagement difficult’. (C 19).
	‘Digital poverty (in terms of access to equipment and IT knowledge/skills) continues to be an issue for us’. (C 5).
	‘Participants were generally receptive of the idea for a remote group, however, there were some issues in clients being able to download Teams to their device’. (C 13).
	‘We needed to get and distribute some devices for our clients to use’. (C 30).
Support needed from family member / carer	‘There have been ongoing difficulties with participants accessing technology and we have relied upon carers to assist, e.g., downloading Teams’. (C 19).
	‘Internet access was and still is key barrier — most participants struggled to use without carer help. Now becoming harder to recruit because family members who were supporting are back at work’. (C 21).
Difficulty in recruiting	‘Sadly, we’'ve found it really hard to get enough people willing to join so haven’'t been able to offer a group’. (C 18).
Staff time commitment	‘Fairly time-consuming setting up, e.g., phoning around, getting emails, sending out pre group info and then sending WebEx invitations’. (C 15).
	‘A lot of time has been taken up making initial phone calls, engaging in 1:1 trial sessions to check access and usability — more time and staff than can be justified for such a small group’. (C 21).
Adapting sessions	‘Initial set-up took a lot of time and sharing of ideas across six localities (timings, risk assessment, adapting activities to suit online presentation)’. (C 21).
	‘[We] Have adapted [the] programme as required — found sounds session more difficult as sound quality not so good for some on WebEx. Also chose to do a seated chair exercise to music at beginning rather than singing in some groups’. (C 15).
Staff require IT support	‘When we were ready to get information printed and videos uploaded, could not get the IT support that was needed as this department was inundated by the whole trust’. (C 6).
	‘Staff side requires dual screens’. (C 23).
Missing social aspect	‘Satisfaction not as high as group setting as social stimulation and peer support missed out’. (C 9).
Impact of sensory impairment	‘The negatives include the anxieties of struggling with technology, especially if dealing with sensory impairments like hearing loss… a lot of patients will need face to face in future due to a lack of technology or sensory problems that make online engagement difficult’. (C 19).

### Benefits of Offering Virtual CST Services

This theme is derived from the responses of the 18 services offering vCST. A key advantage reported by six services was that virtual delivery had made it possible for people with dementia to attend who would not normally be able to due to transport issues. Staff stated that this made the groups more accessible for some participants. Staff from six memory clinics reported perceived benefits for participants including engagement with others and forming social connections; both of which are especially important during periods of isolation due to COVID-19 restrictions. CST was described as ‘something to look forward to’, and staff from four services stated that people with dementia and their carers had self-reported improved mood and cognition. Staff from three services also reported that participants had improved their digital confidence through attending the sessions. One service shared that participants now use videoconferencing to speak to family members, which they had not done before attending the vCST sessions.

Staff from two services reported that they had developed new skills and an improved perceived value of their job role. This was attributed to learning to deliver vCST and being able to engage with people therapeutically, especially as this had not been previously possible due to COVID restrictions.

### Challenges of Offering Virtual CST Services

This theme is derived from the responses of 33 services. These were the 18 services offering vCST, as well as the 15 that did not offer vCST — many of whom reflected on the challenges of trying to set up vCST. Despite the improved access for some participants arising from the removal of transport barriers, 15 services reported issues with access to the required technology, including lack of access to the appropriate devices and internet and poor digital literacy. Six services reported a considerable time commitment required from staff or family carers to support participants to join the sessions. Four services reported difficulty in recruiting or retaining participants. To improve access, two services provided tablet-computers for participants who did not have access to their own. The four memory clinics that were delivering CST individually over the telephone may be reflective of participants’ lack of access to technology, low levels of digital literacy, or lack of interest in joining a virtual group. Alongside the time needed to provide support with technology, staff from four services also reported a large time commitment in organising sessions and developing and adapting materials suitable for virtual delivery. Staff in three services also needed IT support or additional computer equipment in order to run the groups.

There were also challenges reported by the carers and people with dementia. Staff from one service fed back that people with dementia and their carers reported that it was a challenge for people with sensory impairments to engage with technology. Another service had received feedback that participants missed the social aspect of the groups, and that the peer support element of the group was lacking.

### Reasons for Not Delivering Virtual CST

Nine of the 15 memory clinics who were not delivering CST virtually provided their reasoning. Some services had limited staff capacity, and others tried to set up vCST groups but were not able to recruit enough people with dementia to take part virtually. Some clinics reported that service users had expressed their preference for face-to-face groups, so virtual groups were not attempted. Another reason for not providing vCST was service user’s limited access to, or lack of knowledge using, digital technology. In some of these cases, individual CST was offered over the telephone or video calling. Another alternative method of delivery from four services was providing ‘CST activity packs’, which could be completed at home by the service user.

## Discussion

The results of this survey suggest that vCST is a feasible and acceptable alternative to face-to-face CST, with the added advantage that it increases accessibility for those who cannot access face-to-face groups due to ongoing COVID-19 restrictions, or issues related to transport or mobility. A limited amount of research on the use of digital technologies in dementia care prior to the pandemic suggests that, despite the barriers, it is both feasible and beneficial to offer online interventions to people with dementia (Burton & O’Connell, 2018; LaMonica et al., 2017). Since the pandemic, a growing number of case studies and feasibility studies have come to this same conclusion (Cheung & Peri, 2021; Dowson et al., 2021; Lai et al., 2020; Masoud et al., 2021).

Our qualitative findings are in keeping with a past review of qualitative research of face-to-face CST groups (Gibbor et al., 2021) which describes participant outcomes including perceived benefits in participants’ mood, confidence and cognition, and a sense of enjoyment of the sessions. These are also reflected in quantitative data from a review of randomised controlled trials of CST (Lobbia et al., 2019), where key outcomes include benefits to participants’ cognitive function and wellbeing. Despite the virtual method of delivery, there still appears to be a benefit for some individuals from engaging with others, but some services did report that participants missed the social aspect in virtual groups, showing that it may be harder to connect with others virtually. It is possible though that the time burden is greater for vCST with the need to adapt sessions and create virtual resources, support people with dementia to use the technology, and organise the videoconferencing invites. However, this could be offset by time and cost savings related to organising transport and an available room. Research on face-to-face CST also found that participant attendance often relies on transport from carers (Gibbor et al., 2021). Virtual groups help to remove this barrier, but there are additional barriers from technology, and our findings suggest that support from carers is still needed to help access virtual groups.

### Digital Exclusion

Existing literature and qualitative feedback from our survey have both recognised the issue of digital poverty. Whilst the scale of digital exclusion throughout the UK and number of ‘internet non-users’ has been steadily declining over recent years, there were still 5.3 million adults within the UK in 2018 who were classed as ‘internet non-users’ (10% of the UK adult population) (Office for National Statistics, 2019). Those over the age of 75 years old made up over half of all internet non-users. Multiple NHS memory services who participated in our survey explained that lack of access to the appropriate digital technology was an issue and prevented service users from taking part in virtual CST groups. Some memory services needed to obtain and distribute devices for their clients to use. Until this issue is resolved, it would need to be considered how lack of access to digital technology may be addressed to ensure that vCST is available to all those with dementia who would benefit from it, not only those with access to digital devices and reliable internet access.

### Digital Literacy

Furthermore, low levels of digital literacy may be a barrier for older adults (Tan et al., 2020) as videoconferencing calls can be complex to set up and require high-level digital skills. One published systematic review (Yi et al., 2021) explored how previous research studies have addressed these issues by providing additional support sessions for participants, prior to their video calls. Research staff assisted with downloading software, conducting a practice run through, and resolving any technical difficulties, either at the participant’s home (Laver et al., 2020) or via the telephone (Lindauer et al., 2017; Moo et al., 2020). To minimise discomfort and confusion, vCST facilitators could provide service users with simple instructions ahead of their session and ensure that a back-up plan is agreed together (such as reverting to a telephone call should either experience any technical difficulties). As observed in previous literature (Gately et al., 2021), it would be important to establish the perceived technical competence and confidence of the person with dementia beforehand, and then provide the necessary support, training, and reassurance, as required.

### Sensory Impairment

As well as this, sensory impairment is highly prevalent in older adults and could potentially be a barrier to accessing vCST. Around 12 million adults within the UK have a hearing impairment; 40% of over 50-year-olds have a hearing loss, which rises to more than 70% for those over the age of 70 (RNID, 2018). For visual impairment within the UK, 1 in 5 people aged 75 and over, and 1 in 2 people aged 90 and over, are living with sight loss (Office for National Statistics, 2015; Pezzullo et al., 2018). Therefore, it should be considered how vCST could be adapted so that those with sensory impairment are not excluded or denied access. For those with hearing impairment, automatic speech recognition has been made available through various videoconferencing platforms (McKee et al., 2020) and could be used throughout vCST. Facilitators could encourage service users to wear their hearing aids, should they require them, and further assist by integrating headsets, and communicating slowly and clearly. Non-verbal communications, such as displaying pictures throughout sessions, could be used to enable understanding and build rapport (Gately et al., 2021). For those with visual impairment, facilitators could use verbal descriptions and not rely solely on facial expressions and hand gestures for communication (Yi et al., 2021). For both, the environment for the facilitator and service users should be considered (appropriate lighting, limiting background noise) as well as practical considerations to prevent fatigue, such as keeping sessions focused, not too lengthy, and offering breaks.

### Future Research

Future research should look to address the time commitment required for preparing vCST sessions and reduce the duplication of efforts by publishing a standardised vCST protocol for service providers. This approach would then allow efficacy data on vCST to be collected. It would be important for research to formally evaluate whether the well-documented effects of group CST can be replicated though virtual delivery.

### Limitations

Of the 41 responses, 33 were suitable for analysis, and a number of those did not provide in-depth feedback to the open-ended questions. It is also important to highlight that the feedback from those with dementia and their carers has been reported by the facilitators who answered our survey. These qualitative findings are based on informal feedback received from the people with dementia and their carers and could be influenced by facilitator interpretation or perception.

The response rate of memory clinics on the MSNAP mailing list was 22% (19/85). We cannot ascertain the exact response rate from the CHAIN newsletter, as many of the 564 CHAIN members work within general health, social care, or research, and not exclusively in memory clinics. A comparable survey on CST provision prior to the pandemic received responses from 57/186 services (response rate 30.7%). The authors reported that 41 responses were initially received, at which point follow-up contact was made via phone and email, which resulted in 16 more submissions (Holden et al., 2021). Our survey was only promoted through email newsletters. We may have obtained a higher response rate if individual memory clinics had been contacted directly via email or telephone.

A lack of staffing capacity during the COVID-19 pandemic may have contributed to our lower response rate. Our response rate is comparable to that of an April 2020 survey assessing memory clinic provision for people from diverse ethnic backgrounds in England and Wales, which received responses from 20/213 memory services (Brown et al., 2021).

Finally, we contacted a sub-sample of NHS memory clinics, which may not be representative of all clinics. This is especially apparent for those accredited to MSNAP, where delivery of CST is mandated as an accreditation standard (Royal College of Psychiatrists, 2016). Furthermore, memory clinics delivering CST virtually may have been more inclined to take part in the survey, whereas those not providing this service may have been less likely to respond. This could mean that clinics delivering CST virtually are overrepresented in our sample. Therefore, our sample may not accurately reflect service provision across NHS memory clinics.

## Conclusions

There have been many challenges in adapting services, particularly for vulnerable adults, during the COVID-19 pandemic. Delivering CST virtually has been a feasible alternative to face-to-face services during the restrictions, but the results suggest that vCST sessions should not completely replace in-person groups in the future. A hybrid model of offering separate in-person and online groups may be the best recommendation. This approach increases accessibility for all and allows service users to choose the method of delivery that suits them best.

Future research should explore the efficacy of vCST, adapt and disseminate materials for virtual CST, and explore which videoconferencing platform is most suitable for vCST. A larger scale, follow-up survey would enable exploration of changing patterns of CST delivery. It would be interesting to explore who may be excluded from digital interventions, due to digital poverty or lack of technological knowledge, and whether differs by geographical region, socioeconomic status, or other personal characteristics. This would be vital in order to make vCST accessible to all those who would benefit from the intervention.

## Supplementary Information

Below is the link to the electronic supplementary material.

Supplementary file1 (DOCX 15 KB)

## Abbreviations

CHAIN: Contacts, Help, Advice & Information Network
CST: Cognitive Stimulation Therapy
vCST: Virtual Cognitive Stimulation Therapy
MSNAP: Memory Services National Accreditation Programme
NHS: National Health Service
NICE: National Institute for Health and Care Excellence
