# Supplementary material for: Is Virtual Cognitive Stimulation Therapy the Future for People with Dementia? An Audit of UK NHS Memory Clinics During the COVID-19 Pandemic
Source: J Technol Behav Sci. 2023 Feb 24:1–8. Online ahead of print. doi: 10.1007/s41347-023-00306-5 (PMC9950015; doi:10.1007/s41347-023-00306-5)
Supplement: Supplementary file 1 — Supplementary file1 (DOCX 15 KB) [file 41347_2023_306_MOESM1_ESM.docx]

# Appendix 1

**Survey questions**

**Brief Virtual CST survey**

1. What is the name of your Memory Clinic and the NHS trust that the Memory Clinic falls under (e.g. Havering Memory Clinic, North East London NHS Foundation Trust). Please spell out any acronyms.
2. Prior to the COVID-19 pandemic, were you offering Cognitive Stimulation Therapy (CST) within your service?

Yes -> go to question 3

No-> go to question 4

1. Please briefly describing what you were offering to people with dementia, for example 14 twice weekly sessions; 10 weekly sessions.
2. Since the COVID-19 pandemic, have you been offering any CST services virtually?

Yes -> go to question 5

No-> go to question 9

1. Please describe what you are offering, for example: individual or group sessions, platform (Teams, Zoom, etc), rough numbers of people per group, frequency and number of sessions.
2. What has been your experience of virtual CST services? For example, has it been easy to set up, have people with dementia seemed keen to join, have there been issues with participants’ access to the internet or suitable devices
3. Have you gathered any feedback from people with dementia and their carers? Please briefly summarise any feedback below.
4. Once face-to-face services fully resume, how do you plan to continue with virtual CST services?
5. Face-to-face only
6. Virtual only
7. Mixed virtual and face-to-face
8. Please provide any additional comments you wish to share (but please don't include any personal or contact information).
